# Editorial: The Genetic Causes Underlying Immune Mediated Disease: A Focus on Autoimmunity and Cancer

**DOI:** 10.3389/fgene.2022.889160

**Published:** 2022-03-25

**Authors:** Silvia Jiménez-Morales, Xianwen Ren, Michael Dean

**Affiliations:** ^1^ Laboratory of Cancer Genomics, Instituto Nacional de Medicina Genómica, Mexico, Mexico; ^2^ Beijing Advanced Innovation Center for Genomics, and School of Life Sciences, Peking University, Beijing, China; ^3^ Laboratory of Translational Genomics, Division of Cancer Epidemiology and Genetics, National Cancer Institute, Rockville, MD, United States

**Keywords:** immune-mediated disease, systemic lupus erythematosus, neuromyelitis optica spectrum disorder, Ewing Sarcoma, hormone-related cancer, genomics

Immune-mediated diseases (IMD) are a complex group of highly disabling chronic entities resulting from the abnormal activity of the immune cells and affect up to 10% of the population worldwide (El-Gabalawy et al., 2010; Reale et al., 2018). These diseases display a broad clinical and biological heterogeneity, characterized by a marked inflammatory response, increased attack to self-molecules, or loss of the ability to recognize and fight against tumor cells (He et al., 2022). Systemic lupus erythematosus (SLE) and rheumatoid arthritis (RA) are the prototypes of IMD regarding autoimmunity and inflammation, respectively. But diabetes type 1, asthma, celiac disease, inflammatory bowel disease, idiopathic thrombocytopenic purpura, and cancer are also considered IMD (He et al., 2022). The causes of IMD are multifactorial, attributed to environmental factors, living conditions, microbiota along with individual intrinsic factors. Environmental elements include viruses, bacteria, radioactivity, environmental pollution, and diet. The host components include heredity, gender, race, and age (Scott et al., 2010; Harry et al., 2018). Among the genetic causes, single nucleotide polymorphisms (SNPs) are widely studied as susceptibility genetic risk, and protective factors, and some of them are evaluated as potential therapeutic targets.

In two original research articles, SLE and neuromyelitis optica spectrum disorder (NMOSD) diseases were studied to identify SNPs associated with the susceptibility to these diseases in Mexican populations (Juárez-Vicuña et al.; Rosas-Madrigal et al.). On one hand, Juárez-Acuña et al., studying 439 SLE patients and 358 healthy blood donors found that rs12279860 variant located in interferon lambda 3/4 (*IFNλ3/4*) gene does not confer risk to SLE. However, it was observed that rs12279860 genotypes correlate with the expression of 2′-5′-oligoadenylate synthetase-like (OASL) (Juárez-Vicuña et al.), a critical antiviral factor. The molecular mechanism underlying this finding remains unknown.

On the other hand, Rosas-Madrigal et al. performed a case-control study investigating the SNP associations in HLA class II and tymopoyetin (*TMPO*) genes. The authors report for the first time an association between *TMPO-*rs17028450 and the risk to NMOSD. The rs17028450 variant was also explored in multiple sclerosis and SLE Mexican cases, but no association was found. This study adds to the knowledge regarding the genetic background of NMOSD.

To investigate the immune microenvironment in Ewing Sarcoma (ES) patients for immune-related gene signatures identification, Zhou et al. used mRNA-seq expression data retrieved from the NCBI Gene Expression Omnibus (GEO) repository. They found that the immune-related genes *FMO2*, *GLCE*, *GPR64*, *IGFBP4*, *LOXHD1*, *PBK*, *SNAI2*, *SPP1*, *TAPT1-AS1*, and *ZIC2* comprises an immune signature associated with prognosis in ES and suggest that these genes are potential biomarkers, putting them in line to be validated by other researcher groups.

In addition, a review article displays state of the art information relating to hormone-related cancer and autoimmune diseases (Losada-García et al.). Lozada-García et al. point out inflammation as critical and determinant to cancer and IMD development. Those sex hormones involved in prostate cancer could have a role in the chronic inflammation and autoimmunity, increasing the risk of developing SLE and AR. This work raises questions on the relation among IMD, cancer risk and evolution, and treatment implications.

Even with the diversity of the clinical presentation of IMD, all of them have inflammation a common pathway. However, we need to learn more about the shared causes, the genetic factors involved in their development, and the identification of biomarkers as potential therapeutic targets.
